# SATB2 expression in hematolymphoid neoplasms

**DOI:** 10.1007/s12308-023-00543-w

**Published:** 2023-04-03

**Authors:** Gerald C. Tiu, Yasodha Natkunam, Sebastian Fernandez-Pol

**Affiliations:** 1 Department of Pathology, Stanford University School of Medicine, Stanford, CA 94305 USA; 2 Department of Pediatrics, University of California San Francisco, San Francisco, CA 94158 USA

**Keywords:** SATB2, Immunohistochemistry, Leukemia, Lymphoma

To the Editor:

Special AT-rich binding protein 2 (SATB2) is a DNA-binding protein with important roles in transcriptional regulation and chromatin organization [1]. Immunohistochemical staining for SATB2 has become a widespread and invaluable tool in diagnostic pathology given its role as a sensitive and specific marker for colorectal carcinoma, Merkel cell carcinoma, and tumors with osteoblastic differentiation [2, 3]. Recent studies have reported a pathogenic role of SATB2 in *BCR::ABL*-positive B-cell leukemia [4]. However, to our knowledge, a systematic study of SATB2 expression in hematolymphoid malignancies has not been reported. Here, we investigate SATB2 expression by immunohistochemistry in 241 cases of myeloid and lymphoid neoplasms. We find positive SATB2 expression in rare cases of classic Hodgkin lymphoma (CHL) and mature T- and NK-cell neoplasms but negative expression in the vast majority of hematolymphoid neoplasms tested.

To systematically investigate SATB2 protein expression, immunohistochemical staining for SATB2 (Cell Marque, clone EP281, 1:2 dilution, Leica Bond 3 platform, ER2 retrieval buffer) was performed on 241 cases of hematolymphoid malignancies and 6 cases of control placenta, lymph node, and tonsil tissue in which cases with any expression of SATB2 were counted as positive (Fig. 1a). The vast majority of hematolymphoid neoplasms and control placenta, lymph node, and tonsil tissues tested were negative (218 of 247, 88%). Positive internal control staining was seen in bone marrow osteoblasts, which showed bright 3 + nuclear expression, confirming that the staining protocol worked well in decalcified bone marrow biopsies.

**Fig. 1:**
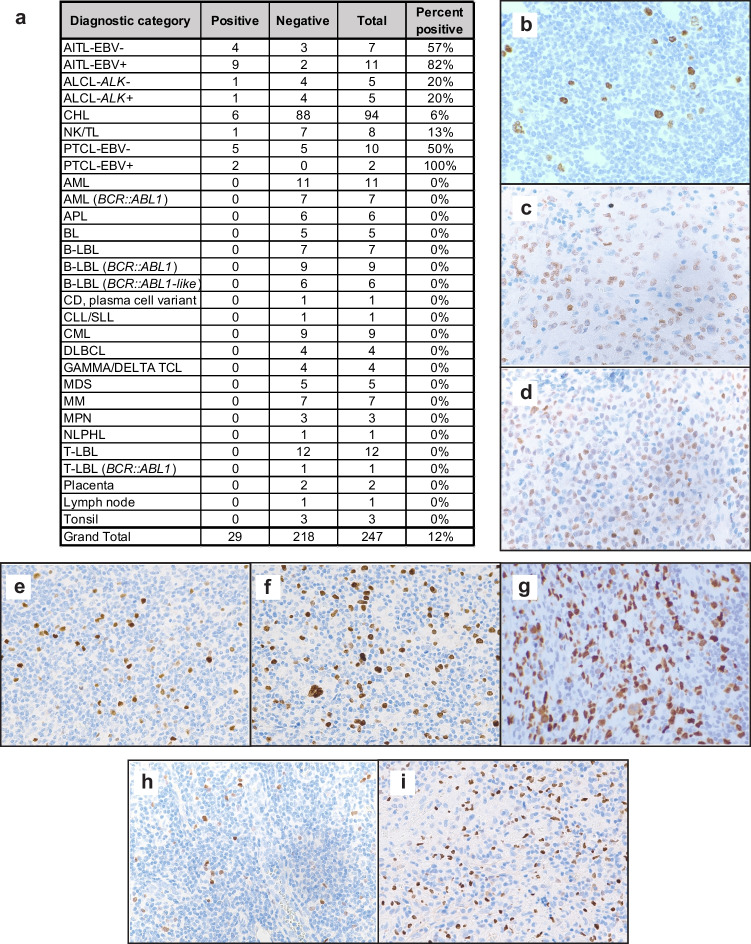
SATB2 protein immunoreactivity in hematolymphoid neoplasms (AITL-EBV- = Angioimmunoblastic T-cell lymphoma, EBV negative; AITL-EBV +  = Angioimmunoblastic T-cell lymphoma, EBV positive; ALCL-*ALK*- = Anaplastic large cell lymphoma, ALK-negative; ALCL-*ALK* +  = Anaplastic large cell lymphoma, ALK-positive; CHL = Classic Hodgkin lymphoma; NK/TL = Extranodal NK/T-cell lymphoma, nasal type; PTCL-EBV- = Peripheral T-cell lymphoma, not otherwise specified, EBV negative; PTCL-EBV +  = Peripheral T-cell lymphoma, not otherwise specified, EBV positive; AML = Acute myeloid leukemia; AML (*BCR::ABL1*) = Acute myeloid leukemia with *BCR::ABL1*; APL = Acute promyelocytic leukemia; BL = Burkitt lymphoma; B-LBL = B-lymphoblastic leukemia; B-LBL (*BCR::ABL1*) = B-lymphoblastic leukemia with *BCR::ABL1*; B-LBL (*BCR::ABL1-*like) = B-lymphoblastic leukemia with *BCR::ABL1*-like; CD = Castleman’s disease; CLL/SLL = Chronic lymphocytic leukemia/small lymphocytic lymphoma; CML = Chronic myeloid leukemia; DLBCL = Diffuse large B-cell lymphoma; GAMMA/DELTA TCL = Gamma delta T-cell lymphoma; MDS = Myelodysplastic syndrome; MM = Multiple myeloma; MPN = Myeloproliferative neoplasm; NLPHL = Nodular lymphocyte predominant Hodgkin lymphoma; T-LBL = T-lymphoblastic leukemia; T-LBL (*BCR::ABL1*) = T-lymphoblastic leukemia with *BCR::ABL1.*).** a**) Table of SATB2 protein immunoreactivity in 241 cases of hematolymphoid neoplasms and 6 control tissues (247 samples total). **b-i**) Representative images of **b**) CHL; **c**) ALCL-*ALK-*; **d**) ALCL-*ALK* + ; **e**) PTCL-EBV- **f**) PTCL-EBV + ; **g**) NK/TL; **h**) AITL-EBV-; **i**) AITL-EBV +

SATB2 expression was restricted to mature T- and NK-cell lymphomas and rare cases of CHL (6 of 94 cases, 6%). As shown in Fig. 1b, the rare cases of CHL showed SATB2 expression in Hodgkin/Reed-Sternberg cells.

Among anaplastic large cell lymphomas (ALCL), 1 of 5 cases of *ALK*-negative ALCL was positive with 1 + SATB2 expression in approximately 50% of the lymphoma cells (Fig. 1c), and 1 of 5 cases of *ALK*-positive ALCL was positive with 1 + expression in approximately 50% of the lymphoma cells (Fig. 1d).

SATB2 was also positive in 5 of 10 cases of EBV-negative peripheral T-cell lymphoma, not otherwise specified (PTCL) (Fig. 1e). Two of the positive cases showed only rare positive cells in the range of 0–1% of the infiltrate with an intensity of 3 + . One case showed 2 + signal in 5% of the cells, and 2 cases showed strong (2–3 +) signal in 75% and 90% of the lymphoma cells. Among EBV-positive PTCL, 2 of 2 cases were positive showing 5% of cells with 2 + expression and 15% of cells with 3 + expression, respectively (Fig. 1f). In addition, 1 of 8 extranodal NK/T-cell lymphoma, nasal type (NK/TL) was positive with 80% of cells showing 3 + expression (Fig. 1g).

Eighteen angioimmunoblastic T-cell lymphoma (AITL) samples were stained. Among the 7 EBV-negative AITL cases assayed, 4 showed rare SATB2-positive cells (0–1% range, 2–3 + intensity) (Fig. 1h). Among the 11 cases with EBV + cells (either in a secondary EBV + B-cell proliferation or without a B-cell proliferation), 9 showed SATB2-positive cells (Fig. 1i). Five of these 9 cases showed SATB2 expression in the range of 0–1% of the infiltrate with 2–3 + intensity. The remaining 4 cases showed 5–30% SATB2-positive cells at 1–3 + intensity.

We next turned to publicly available RNA-Seq data from the Cancer Cell Line Encyclopedia/Cancer Dependency Map (CCLE/DepMap) to investigate messenger RNA (mRNA) expression of *SATB2* in cell lines derived from various hematological malignancies (Fig. 2) [5]. In agreement with our immunohistochemistry findings, ALCL, cutaneous T-cell lymphoma (CTCL), and Hodgkin lymphoma (HL) cell lines demonstrated increased *SATB2* mRNA expression. A subset of these cell lines showed *SATB2* mRNA levels up to what is observed in cell lines derived from colorectal adenocarcinoma, which is known to express high levels of *SATB2* (Fig. 2) [2]. Of note, high levels of *SATB2* mRNA expression are also observed in cell lines derived from CML, acute promyelocytic leukemia (APL), and a subset of acute myeloid leukemia (AML).

**Fig. 2:**
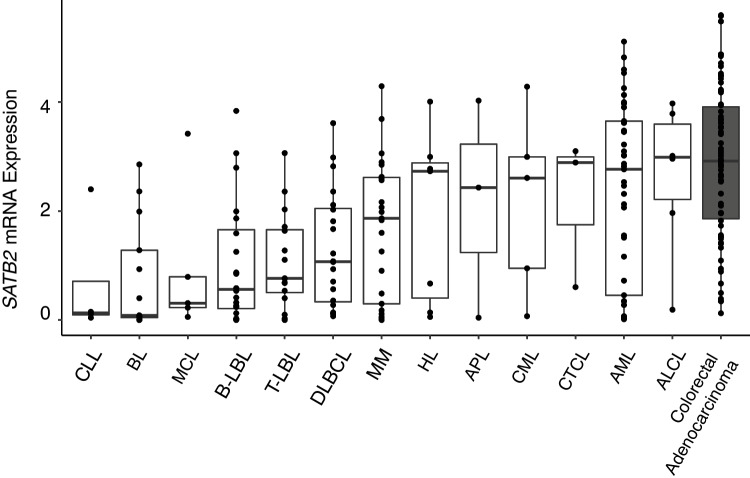
SATB2 mRNA expression in cell lines derived from hematologic neoplasms (CLL = Chronic lymphocytic leukemia; BL = Burkitt lymphoma; MCL = Mantle cell lymphoma; B-LBL = B lymphoblastic leukemia; T-LBL = T lymphoblastic leukemia; MM = Multiple myeloma; HL = Hodgkin lymphoma; APL = Acute promyelocytic leukemia; CML = Chronic myeloid leukemia; CTCL = Cutaneous T-cell lymphoma; AML = Acute myeloid leukemia; ALCL = Anaplastic large cell lymphoma). RNA-Seq mRNA expression is expressed as transcripts per million + 1 (TPM + 1). Data is derived from the Cancer Cell Line Encyclopedia/Cancer Dependency Map [5]

Overall, through systematic immunohistochemical analysis of SATB2 expression in a large cohort of hematolymphoid neoplasms, we demonstrate positive SATB2 protein staining in rare cases of CHL (6 of 94, 6%) and in subsets of mature T- and NK-cell neoplasms: AITL-EBV- (4 of 7, 57%), AITL-EBV + (9 of 11, 82%), ALCL-*ALK*- (1 of 5, 20%), ALCL-*ALK* + (1 of 5, 20%), PTCL-EBV- (5 of 10, 50%), PTCL-EBV + (2 of 2, 100%), and NK/TL (1 of 8, 13%). The overall immunohistochemical findings are corroborated by publicly available CCLE RNA sequencing data from hematolymphoid neoplasm cell lines (Fig. 2). Although our immunohistochemical case series and the CCLE RNA-Seq cell line data are in general agreement, there are some notable differences. A subset of cell lines derived from CML, APL, and AML show high SATB2 mRNA expression whereas we observed no SATB2 protein expression by immunohistochemistry from patient tissue samples. In addition, no SATB2 IHC expression was seen in nine *BCR::ABL1*-positive B-LBL in our dataset despite reported expression of *SATB2* transcript in a prior study [4]. Furthermore, seven *BCR::ABL1*-positive AML cases and one *BCR::ABL1*-positive T-LBL case were negative for SATB2. Our findings suggest that SATB2 protein expression is not common in *BCR::ABL1*-positive neoplasms. The differences between the prior mRNA data from the above studies and our data may be due to differences in sampling, post-transcriptional gene regulation, assay sensitivity between IHC and RNA-based methods, or biological differences between cultured cell lines and in situ tissue samples. Overall, this work provides a groundwork for studying SATB2 expression in hematolymphoid neoplasms.

## Data Availability

Data related to this study are available upon reasonable request.

## Declarations

### Data sharing statement

The datasets generated during and/or analyzed during the current study are available from the corresponding author on request.
